# Procalcitonin levels in patients undergoing left ventricular assist device implantation

**DOI:** 10.1186/cc14141

**Published:** 2015-03-16

**Authors:** M Holek, J Kettner, J Franeková, A Jabor

**Affiliations:** 1 Institute for Clinical and Experimental Medicine, Prague, Czech Republic

## Introduction

Procalcitonin (PCT) is used for diagnosis of a bacterial infection. Several works described nonspecific elevation of PCT after cardiac surgery with cardiopulmonary bypass (CPB) caused by systemic inflammatory response syndrome (SIRS) with various cutoff values for the presence of the infection (0.47 to 2.47 μg/l). However, in patients undergoing left ventricular assist device (LVAD), implantation data about PCT dynamics are lacking.

## Methods

PCT levels in 25 patients indicated for LVAD were prospectively assessed before surgery and during the postoperative period (days 1, 2, 14 and 30). Values were compared according to the presence of infectious complications (IC) and non-infectious complications such as acute renal failure (ARF) defined as injury by RIFLE criteria or necessity of right ventricular assist device (RVAD). Data were also analyzed using combined endpoints A (ARF, RVAD) and B (IC, ARF, RVAD). Values are presented as median with interquartile range (in μg/l).

## Results

PCT levels were low before surgery (0.16, 0.10 to 0.35), increased significantly within the first (5.72, 2.18 to 9.75; *P *< 0.001) and second (5.94, 2.54 to 11.99; *P *< 0.001) day after operation and decreased on the 14th (0.27, 0.11 to 0.74) and 30th (0.10, 0.06 to 0.19) day. There was no significant difference in PCT values between patients with or without IC as well as with or without RVAD. ARF increased PCT level significantly only 14 days after LVAD implantation (0.68, 0.37 to 1.65 vs. 0.15, 0.11 to 0.34; *P *= 0.015). Subjects with endpoint A had significantly higher PCT values on the second (19.53, 5.66 to 63.12 vs. 3.95, 2.33 to 8.85; *P *= 0.033), 14th (0.55, 0.31 to 1.44 vs. 0.15, 0. to 0.34; *P *= 0.020) and 30th (0.19, 0.11 to 0.29 vs. 0.08, 0.05 to 0.13; *P *= 0.016) day after operation. Patients with endpoint B had significantly elevated PCT levels 2 (11.99, 3.23 to 24.16 vs. 3.95, 2.54 to 7.39; *P *= 0.027) and 14 (0.55, 0.28 to 0.90 vs. 0.13, 0.09 to 0.23; *P *= 0.005) days after surgery.

## Conclusion

PCT levels in patients undergoing LVAD implantation rise significantly in the first 2 days after surgery. Interestingly, this elevation is much higher than after routine cardiac surgery with CPB. Recent works suggest that PCT concentrations are affected by SIRS caused by contact with a nonphysiological surface. In the case of LVAD this immunological stimulation is long lasting and even more potent with additional RVAD or ARF treated with renal replacement therapy. In accordance with this hypothesis, our data show that the ability of PCT to detect infectious complication in LVAD patients is limited and its concentrations more probably correlate with postoperative complications in general.

